# Supplementary material for: Spotting the Targets of the Apospory Controller TGS1 in Paspalum notatum
Source: Plants (Basel). 2022 Jul 26;11(15):1929. doi: 10.3390/plants11151929 (PMC9332697; doi:10.3390/plants11151929)
Supplement: Supplementary file 1 [file plants-11-01929-s001.zip › Supplementary Data S1.pdf]

**Supplementary Data S1: Primer design for PCR amplification of the splice variants carrying introns (unprocessed forms).** The left primer was always located inside the intron. The putative GT/AG consensus splicing sites were marked in yellow.

## i10779

Homolog to three isotigs in the sexual database (SEXisotig03671, SEXisotig03672, and SEXisotig03673) and one isotig in the apomictic one (APOisotig23969)

>SEXisotig03671  gene=isogroup00552  length=1857  numContigs=5
GTTGGCAATCGCTTCCGTGCAccccTTTCCTATACagtgtccccgggatgatCGTTCAT
CAGTCACCACCGACAACGACAAGGTACAGCaaaTGGAggTTGAGGTTCGCAGAAACAGAA
AGAaCAAAACACGGTAAaGCAAATGgCGAGATTTTATTCGACCGgTCGTATATAACCCGT
GAGCGATGAAACTGACAGCAAACAGTTTAAGCAAGGACGCAGACATGACATGGCATGGAA
TTATTGGGACGGATTTGACGGGGAGCCAACGATTTGTGTGGTAGACGCGTCATCCAACAT
CTCTTCATCAATCACAAGGGGCACTCTAGCAGGTGGCCCTGaTCTTTGAGCGACTGGACG
GCGTCGTCGAACATCTCATGCACCCCTCTGAACTTGATGCCCAGTGCCTGCAGCTTCGAC
GTGTTCAGCTCGTACGACAGCTTGCCGTAAGGGTTGTTCAGTCTCCGAGGTATGGGGAAG
ATGGGGTATCGTTTGGCGAGCAAGGAGACGAGCTCGTCGTTGTCCAGCACCACGGAGCTG
CACAGGTACCTCCCGGCGGCCTCGGGGGCCTCGTACACCAGGATGTGGCTGCTTGtAACG
TCGTCGATGTGAACGTATCCCATTCTTCCGTAAGAACTGAACCTTGCCGTGTCACCTGAA
AAGAGAGGGGACATACTCTGTGTAaTGTTAGGGCTCGCATGATAGgCTGAGTGCCTGCCG
TCAGAGTTAACATGTGACAGACAGACAGAGCTAAATGGTGATATATATATGGAGATCGAA
GGAGCATATATAGGCACCTTGGAATAAGCCAAGGACGTCTGAAGCCGTCACACATAACTC
GTGGGATAAGCTGGGACCGACCACGAACGATGGAAGAACAGTCACTAGGTCAATGTTGTT
CTCCTTGGCAAACTCCCATGCTGCTTtCTCTGCCAATACCTTCGCCAGGGCATACCATAG
CTTTGGATGAAAACAAACAGGCGAAGGCAGAACACCATtATATTCTTCAGATCACGGTTT
CAGAACACATGGTTATGCAAAaGCAaGgTGGgTAATGgATtGTtCGATGTATCACCTGCA
TCTTTTCACAGAGTGGCACAGAGCTCCATGTTGTTTCGTCCAGTGAGATATTTGGCTGAA
CATCGTCCCTAATCCTCACAGCAGATGACGAAGATGTGAGAACAACCCTTTTCAGAAAGG
GGTTCTTCTTGCACGACCTTAGCACGTTCAGTGTGCCGTTTACTGCAGGAACGAGAGTTT
CCTCCTGCAGCATGGaTATCACACATGAAAaCAGTTTAAATTCAGAGTAGATGCATTTCA
aCAGAAGGAAACCAGTATTtGAACGTCGACTGCAACTTTTAAaGCTAAAATAAAAATAGC
ATATCGATACAAAAAAATCTGAATGAAAGACTGTAGCAGGATGCATGATTCAGATGCATA
TGCAGCATGATGGTGATGGCGGATGAAAGAGCGAACTGTTAAAAGCAAATGCTGCATGCC
TTGCTACTAGAATCAGATTTAGCGAGGACAGGCGATGCGGTGTGGAAAACGCCATCACAA
TCCATCACGGCaTCgTCAAaGcTCCCTTCCTCCAACAGaTCaGCTCGCACaATCTGCAGC
CTCTCtTTAGcAcCAgGTaATTTCCAAAGGTGTGCAACTTTTTGCTGATTTCCTGGGTCT
CTGATAGTCCCTACCACATGATATCCGGACTCGAGAAGCCGTTTGATGAGCCAAGAGGCA
ATGAAACCTGAAGCACCGGTTACGCAAACTTTGCCTTTGCTTGAGCTCACCATTTTtCTC
AGTCAGTTGCAAGTAATGCTGATTTCctGGGTCtCTGAtAgTCCcTACcACATGATA

Putative intron 1
LEFT PRIMER: CTCGCATGATAGgCTGAGTG
RIGHT PRIMER: ATTGGCAGAGaAAGCAGCAT

Putative intron 2
LEFT PRIMER: GCGAAGGCAGAACACCATtA
RIGHT PRIMER: AAGGTCGTGCAAGAAGAACC

Putative intron 3
LEFT PRIMER: TCGACTGCAACTTTTAAaGCTAA
RIGHT PRIMER: GGAAATtACcTGgTgCTAAaGAGA

**Primers designed on internal sequences of the putative intron 1**

---

PRIMER PICKING RESULTS FOR SEXisotig03671  gene=isogroup00552  length=1857  numContigs=5

No mispriming library specified
Using 1-based sequence positions

| OLIGO | start | len | tm | gc% | any | 3' | seq |
|---|---|---|---|---|---|---|---|
| LEFT PRIMER | 694 | 20 | 59.57 | 55.00 | 4.00 | 0.00 | CTCGCATGATAGgCTGAGTG |
| RIGHT PRIMER | 937 | 20 | 59.98 | 45.00 | 3.00 | 3.00 | ATTGGCAGAGaAAGCAGCAT |

SEQUENCE SIZE: 1857
INCLUDED REGION SIZE: 1857

PRODUCT SIZE: 244, PAIR ANY COMPL: 4.00, PAIR 3' COMPL: 2.00
TARGETS (start, len)*: 721,147

1 GTTGGCAATCGCTTCCGTGCAccccTTTCCTATACagtgtccccgggatgatCGTTCAT

61 CAGTCACCACCGACAACGACAAGGTACAGCaaaTGGAggTTGAGGTTCGCAGAAACAGAA

121 AGAaCAAAACACGGTAAaGCAAATGgCGAGATTTTATTCGACCGgTCGTATATAACCCGT

181 GAGCGATGAAACTGACAGCAAACAGTTTAAGCAAGGACGCAGACATGACATGGCATGGAA

241 TTATTGGGACGGATTTGACGGGGAGCCAACGATTTGTGTGGTAGACGCGTCATCCAACAT

301 CTCTTCATCAATCACAAGGGGCACTCTAGCAGGTGGCCCTGaTCTTTGAGCGACTGGACG

361 GCGTCGTCGAACATCTCATGCACCCCTCTGAACTTGATGCCCAGTGCCTGCAGCTTCGAC

421 GTGTTCAGCTCGTACGACAGCTTGCCGTAAGGGTTGTTCAGTCTCCGAGGTATGGGGAAG

481 ATGGGGTATCGTTTGGCGAGCAAGGAGACGAGCTCGTCGTTGTCCAGCACCACGGAGCTG

541 CACAGGTACCTCCCGGCGGCCTCGGGGGCCTCGTACACCAGGATGTGGCTGCTTGtAACG

601 TCGTCGATGTGAACGTATCCCATTCTTCCGTAAGAACTGAACCTTGCCGTGTCACCTGAA

661 AAGAGAGGGGACATACTCTGTGTAaTGTTAGGGCTCGCATGATAGgCTGAGTGCCTGCCG
>>>>>>>>>>>> >> >> >> >>

721 TCAGAGTTAACATGTGACAGACAGACAGAGCTAAATGGTGATATATATATGGAGATCGAA
*********************************************************************************************************

781 GGAGCATATATAGGCACCTTGGAATAAGCCAAGGACGTCTGAAGCCGTCACACATAACTC
*********************************************************************************************************

841 GTGGGATAAGCTGGGACCGACCACGAACGATGGAAGAACAGTCACTAGGTCAATGTTGTT
***************************

901 CTCCTTGGCAAACTCCCATGCTGCTTtCTCTGCCAATACCTTCGCCAGGGCATACCATAG
<<<<<<<<<<<<<< <<<< < <<

961 CTTTGGATGAAAACAAACAGGCGAAGGCAGAACACCATtATATTCTTCAGATCACGGTTT

1021 CAGAACACATGGTTATGCAAAaGCAaGgTGGgTAATGgATtGTtCGATGTATCACCTGCA

1081 TCTTTTCACAGAGTGGCACAGAGCTCCATGTTGTTTCGTCCAGTGAGATATTTGGCTGAA

1141 CATCGTCCCTAATCCTCACAGCAGATGACGAAGATGTGAGAACAACCCTTTTCAGAAAGG

1201 GGTTCTTCTTGCACGACCTTAGCACGTTCAGTGTGCCGTTTACTGCAGGAACGAGAGTTT

1261 CCTCCTGCAGCATGGaTATCACACATGAAAaCAGTTTAAATTCAGAGTAGATGCATTTCA

1321 aCAGAAGGAAACCAGTATTtGAACGTCGACTGCAACTTTTAAaGCTAAAATAAAAATAGC

1381 ATATCGATACAAAAAAATCTGAATGAAAGACTGTAGCAGGATGCATGATTCAGATGCATA

1441 TGCAGCATGATGGTGATGGCGGATGAAAGAGCGAACTGTTAAAAGCAAATGCTGCATGCC

1501 TTGCTACTAGAATCAGATTTAGCGAGGACAGGCGATGCGGTGTGGAAAACGCCATCACAA

1561 TCCATCACGGCaTCgTCAAaGcTCCCTTCCTCCAACAGaTCaGCTCGCACaATCTGCAGC

1621 CTCTCtTTAGcAcCAgGTaATTTCCAAAGGTGTGCAACTTTTTGCTGATTTCCTGGGTCT

1681 CTGATAGTCCCTACCACATGATATCCGGACTCGAGAAGCCGTTTGATGAGCCAAGAGGCA

1741 ATGAAACCTGAAGCACCGGTTACGCAAACTTTGCCTTTGCTTGAGCTCACCATTTTtCTC

1801 AGTCAGTTGCAAGTAATGCTGATTTCctGGGTCtCTGAtAgTCCcTACcACATGATA

KEYS (in order of precedence):
****** target
>>>>>> left primer
<<<<<< right primer

**Primers designed on internal sequences of the putative intron 2**

---

PRIMER PICKING RESULTS FOR SEXisotig03671 gene=isogroup00552 length=1857 numContigs=5

No mispriming library specified
Using 1-based sequence positions

| OLIGO | start | len | tm | gc% | any | 3' | seq |
|---|---|---|---|---|---|---|---|
| LEFT PRIMER | 981 | 20 | 61.17 | 50.00 | 2.00 | 2.00 | GCGAAGGCAGAACACCATtA |
| RIGHT PRIMER | 1220 | 20 | 59.33 | 50.00 | 6.00 | 2.00 | AAGGTCGTGCAAGAAGAACC |

SEQUENCE SIZE: 1857
INCLUDED REGION SIZE: 1857

PRODUCT SIZE: 240, PAIR ANY COMPL: 3.00, PAIR 3' COMPL: 0.00
TARGETS (start, len)*: 1021,180

1 GTTGGCAATCGCTTCCGTGCAccccTTTCCTATACagtgtccccgggatgatCGTTCAT

61 CAGTCACCACCGACAACGACAAGGTACAGCaaaTGGAggTTGAGGTTCGCAGAAACAGAA

121 AGAaCAAAACACGGTAAaGCAAATGgCGAGATTTTATTCGACCGgTCGTATATAACCCGT

181 GAGCGATGAAACTGACAGCAAACAGTTTAAGCAAGGACGCAGACATGACATGGCATGGAA

241 TTATTGGGACGGATTTGACGGGGAGCCAACGATTTGTGTGGTAGACGCGTCATCCAACAT

301 CTCTTCATCAATCACAAGGGGCACTCTAGCAGGTGGCCCTGaTCTTTGAGCGACTGGACG

361 GCGTCGTCGAACATCTCATGCACCCCTCTGAACTTGATGCCCAGTGCCTGCAGCTTCGAC

421 GTGTTCAGCTCGTACGACAGCTTGCCGTAAGGGTTGTTCAGTCTCCGAGGTATGGGGAAG

481 ATGGGGTATCGTTTGGCGAGCAAGGAGACGAGCTCGTCGTTGTCCAGCACCACGGAGCTG

541 CACAGGTACCTCCCGGCGGCCTCGGGGGCCTCGTACACCAGGATGTGGCTGCTTGtAACG

601 TCGTCGATGTGAACGTATCCCATTCTTCCGTAAGAACTGAACCTTGCCGTGTCACCTGAA

661 AAGAGAGGGGACATACTCTGTGTAaTGTTAGGGCTCGCATGATAGgCTGAGTGCCTGCCG

721 TCAGAGTTAACATGTGACAGACAGACAGAGCTAAATGGTGATATATATATGGAGATCGAA

781 GGAGCATATATAGGCACCTTGGAATAAGCCAAGGACGTCTGAAGCCGTCACACATAACTC

841 GTGGGATAAGCTGGGACCGACCACGAACGATGGAAGAACAGTCACTAGGTCAATGTTGTT

901 CTCCTTGGCAAACTCCCATGCTGCTTtCTCTGCCAATACCTTCGCCAGGGCATACCATAG

961 CTTTGGATGAAAACAAACAGGCGAAGGCAGAACACCATtATATTCTTCAGATCACGGTTT
>>>> >>>>> >>>>>> >> > >>

1021 CAGAACACATGGTTATGCAAAaGCAaGgTGGgTAATGgATtGTtCGATGTATCACCTGCA
****************************************************************************************************

1081 TCTTTTCACAGAGTGGCACAGAGCTCCATGTTGTTTCGTCCAGTGAGATATTTGGCTGAA
********************************************************************************************************

1141 CATCGTCCCTAATCCTCACAGCAGATGACGAAGATGTGAGAACAACCCTTTTCAGAAAGG
************************************************************

1201 GGTTCTTCTTGCACGACCTTAGCACGTTCAGTGTGCCGTTTACTGCAGGAACGAGAGTTT
<<<<<<< <<<<<< <<<< < <<

1261 CCTCCTGCAGCATGGaTATCACACATGAAAaCAGTTTAAATTCAGAGTAGATGCATTTCA

1321 aCAGAAGGAAACCAGTATTtGAACGTCGACTGCAACTTTTAAaGCTAAAATAAAAATAGC

1381 ATATCGATACAAAAAAATCTGAATGAAAGACTGTAGCAGGATGCATGATTCAGATGCATA

1441 TGCAGCATGATGGTGATGGCGGATGAAAGAGCGAACTGTTAAAAGCAAATGCTGCATGCC

1501 TTGCTACTAGAATCAGATTTAGCGAGGACAGGCGATGCGGTGTGGAAAACGCCATCACAA

1561 TCCATCACGGCaTCgTCAAaGcTCCCTTCCTCCAACAGaTCaGCTCGCACaATCTGCAGC

1621 CTCTCtTTAGcAcCAgGTaATTTCCAAAGGTGTGCAACTTTTTGCTGATTTCCTGGGTCT

1681 CTGATAGTCCCTACCACATGATATCCGGACTCGAGAAGCCGTTTGATGAGCCAAGAGGCA

1741 ATGAAACCTGAAGCACCGGTTACGCAAACTTTGCCTTTGCTTGAGCTCACCATTTTtCTC

1801 AGTCAGTTGCAAGTAATGCTGATTTCctGGGTCtCTGAtAgTCCcTACcACATGATA

**Primers designed on internal sequences of the putative intron 3**

---

PRIMER PICKING RESULTS FOR SEXisotig03671 gene=isogroup00552 length=1857 numContigs=5

No mispriming library specified
Using 1-based sequence positions

| OLIGO | start | len | tm | gc% | any | 3' | seq |
|---|---|---|---|---|---|---|---|
| LEFT PRIMER | 1346 | 23 | 58.46 | 34.78 | 7.00 | 3.00 | TCGACTGCAACTTTTAAaGCTAA |
| RIGHT PRIMER | 1645 | 24 | 60.03 | 41.67 | 6.00 | 0.00 | GGAAATtACcTGgTgCTAAaGAGA |

SEQUENCE SIZE: 1857
INCLUDED REGION SIZE: 1857

PRODUCT SIZE: 300, PAIR ANY COMPL: 4.00, PAIR 3' COMPL: 0.00
TARGETS (start, len)*: 1381,240

```
   1 GTTGGCAATCGCTTCCGTGCAccccTTTCCTATACagtgtccccgggatgatCGTTCAT

  61 CAGTCACCACCGACAACGACAAGGTACAGCaaaTGGAggTTGAGGTTCGCAGAAACAGAA

 121 AGAaCAAAACACGGTAAaGCAAATGgCGAGATTTTATTCGACCGgTCGTATATAACCCGT

 181 GAGCGATGAAACTGACAGCAAACAGTTTAAGCAAGGACGCAGACATGACATGGCATGGAA

 241 TTATTGGGACGGATTTGACGGGGAGCCAACGATTTGTGTGGTAGACGCGTCATCCAACAT

 301 CTCTTCATCAATCACAAGGGGCACTCTAGCAGGTGGCCCTGaTCTTTGAGCGACTGGACG

 361 GCGTCGTCGAACATCTCATGCACCCCTCTGAACTTGATGCCCAGTGCCTGCAGCTTCGAC

 421 GTGTTCAGCTCGTACGACAGCTTGCCGTAAGGGTTGTTCAGTCTCCGAGGTATGGGGAAG

 481 ATGGGGTATCGTTTGGCGAGCAAGGAGACGAGCTCGTCGTTGTCCAGCACCACGGAGCTG

 541 CACAGGTACCTCCCGGCGGCCTCGGGGGCCTCGTACACCAGGATGTGGCTGCTTGtAACG

 601 TCGTCGATGTGAACGTATCCCATTCTTCCGTAAGAACTGAACCTTGCCGTGTCACCTGAA

 661 AAGAGAGGGGACATACTCTGTGTAaTGTTAGGGCTCGCATGATAGgCTGAGTGCCTGCCG

 721 TCAGAGTTAACATGTGACAGACAGACAGAGCTAAATGGTGATATATATATGGAGATCGAA

 781 GGAGCATATATAGGCACCTTGGAATAAGCCAAGGACGTCTGAAGCCGTCACACATAACTC

 841 GTGGGATAAGCTGGGACCGACCACGAACGATGGAAGAACAGTCACTAGGTCAATGTTGTT

 901 CTCCTTGGCAAACTCCCATGCTGCTTtCTCTGCCAATACCTTCGCCAGGGCATACCATAG

 961 CTTTGGATGAAAACAAACAGGCGAAGGCAGAACACCATtATATTCTTCAGATCACGGTTT

1021 CAGAACACATGGTTATGCAAAaGCAaGgTGGgTAATGgATtGTtCGATGTATCACCTGCA
```

```
1081 TCTTTTCACAGAGTGGCACAGAGCTCCATGTTGTTTCGTCCAGTGAGATATTTGGCTGAA

1141 CATCGTCCCTAATCCTCACAGCAGATGACGAAGATGTGAGAACAACCCTTTTCAGAAAGG

1201 GGTTCTTCTTGCACGACCTTAGCACGTTCAGTGTGCCGTTTACTGCAGGAACGAGAGTTT

1261 CCTCCTGCAGCATGGaTATCACACATGAAAaCAGTTTAAATTCAGAGTAGATGCATTTCA

1321 aCAGAAGGAAACCAGTATTtGAACGTCGACTGCAACTTTTAAaGCTAAAATAAAAATAGC
                                   >>>>>>>>>> >>>>>>>> >>>> >> >

1381 ATATCGATACAAAAAAATCTGAATGAAAGACTGTAGCAGGATGCATGATTCAGATGCATA
     **************************************************************************************************

1441 TGCAGCATGATGGTGATGGCGGATGAAAGAGCGAACTGTTAAAAGCAAATGCTGCATGCC
     ****************************************************************************************************

1501 TTGCTACTAGAATCAGATTTAGCGAGGACAGGCGATGCGGTGTGGAAAACGCCATCACAA
     ****************************************************************************************************

1561 TCCATCACGGCaTCgTCAAaGcTCCCTTCCTCCAACAGaTCaGCTCGCACaATCTGCAGC
     *********************************************************

1621 CTCTCtTTAGcAcCAgGTaATTTCCAAAGGTGTGCAACTTTTTGCTGATTTCCTGGGTCT
      <<<<<<<<<< <<<<<<<<<<<<<<<<

1681 CTGATAGTCCCTACCACATGATATCCGGACTCGAGAAGCCGTTTGATGAGCCAAGAGGCA

1741 ATGAAACCTGAAGCACCGGTTACGCAAACTTTGCCTTTGCTTGAGCTCACCATTTTtCTC

1801 AGTCAGTTGCAAGTAATGCTGATTTCctGGGTCtCTGAtAgTCCcTACcACATGATA
```

## i22630

Homolog to one isotig in the sexual database (SEXisotig18567) and two in the apomictic one (APOisotig12813 and APOisotig12814)

```
>APOisotig12814  gene=isogroup04206  length=922  numContigs=2
GAGCTTTCAAATTATCTCTGCTTACAGTAGTGTGGTTAATTAATAACAAGAGCTGGAGCT
GGCTTTGCAGGGACCATTTGAATATCcTGCTAGAAGGAGAAGGCACCGGgAGGCTGCTGA
GATATGACCCAGAAaCAAATACTGCCCATGTCGTTCTCAAAGGCCTCGTCTTCCCAAACG
GCGTGCAGATCTCAGAGGACCAGCAGTTTCTCCTCTTCTCCGAGACAACAAATTGCAGGT
CaaaaaaatGTGAATCGACGCTCATGATCCTCCTTACaGAAGATTATAGAGCAGCCAATG
GCTCCATTGACATGAACAATAATTAATTTCTCCTTGATCGATCAGGATAATGAGGTTCTG
GCTGGAAGGCCCAAGAACGGGCcAGCTAGAGGTGTTCGCGAACCTCCCCGGCTTCCCCGA
CAACGTGCGCTCCAACGGCAAGGGCCAGTTCTGGGTGGCGATCGACTGCTGCCGGACGCC
GGCGCAGGAGGTGTTCGCCAAGCGGCCGTGGCTCCGGTCCGTCTACTTCAAGGTCCCGCT
AACGCTCAAGATGCTCACGAGGAGAGCCGTCACGAGTATGCACACGGTGCTCGCGCTCCT
CGACGGCGAGGGCCGCGTCGTGGACGTGCTCGAGGACCGGGGCCGcGAGGTGATGAAGCT
GGTGAGCGAGGTGCGGGAGGTGGGCGGCAAGCTGTGGATCGGGACAGTGGCGCACAACCA
CATCGCCACGGTGCCCTACCCCTTATTGGACTAGCCACAGCGTGCCATTTCCATTGTTAC
TACTGGCTCCTAGTATTATTATGTGTACTACGTCTATAATGGAGTGTGCGATGACTGGTC
GTGTTCTTGATTGTACGTATTTGGCATAACAATGGACAAACTTCAGATCAGAAATCGAAT
AAAATTACTAGTCATGGTTGCT
```

Putative intron

LEFT PRIMER: CaGAAGATTATAGAGCAGCCAATG

RIGHT PRIMER: GCTCTCCTCGTGAGCATCTT

**Primers designed on internal sequences of the putative intron**

---

PRIMER PICKING RESULTS FOR APOisotig12814  gene=isogroup04206  length=922  numContigs=2

No mispriming library specified
Using 1-based sequence positions
OLIGO start len tm gc% any 3' seq
LEFT PRIMER 277 24 59.46 41.67 4.00 3.00 CaGAAGATTATAGAGCAGCCAATG
RIGHT PRIMER 567 20 59.71 55.00 5.00 0.00 GCTCTCCTCGTGAGCATCTT
SEQUENCE SIZE: 922
INCLUDED REGION SIZE: 922

PRODUCT SIZE: 291, PAIR ANY COMPL: 5.00, PAIR 3' COMPL: 3.00
TARGETS (start, len)*: 301,240

1 GAGCTTTCAAATTATCTCTGCTTACAGTAGTGTGGTTAATTAATAACAAGAGCTGGAGCT

61 GGCTTTGCAGGGACCATTTGAATATCcTGCTAGAAGGAGAAGGCACCGGgAGGCTGCTGA

121 GATATGACCCAGAAaCAAATACTGCCCATGTCGTTCTCAAAGGCCTCGTCTTCCCAAACG

181 GCGTGCAGATCTCAGAGGACCAGCAGTTTCTCCTCTTCTCCGAGACAACAAATTGCAGGT

241 CaaaaaaatGTGAATCGACGCTCATGATCCTCCTTACaGAAGATTATAGAGCAGCCAATG
>>>> >>>>>> >>>>> >>>>> >> > >

301 GCTCCATTGACATGAACAATAATTAATTTCTCCTTGATCGATCAGGATAATGAGGTTCTG
******************************************************************************************************

361 GCTGGAAGGCCCAAGAACGGGCcAGCTAGAGGTGTTCGCGAACCTCCCCGGCTTCCCCGA
**************************************************************************************************************

421 CAACGTGCGCTCCAACGGCAAGGGCCAGTTCTGGGTGGCGATCGACTGCTGCCGGACGCC
**************************************************************************************************************

481 GGCGCAGGAGGTGTTCGCCAAGCGGCCGTGGCTCCGGTCCGTCTACTTCAAGGTCCCGCT
************************************************************

541 AACGCTCAAGATGCTCACGAGGAGAGCCGTCACGAGTATGCACACGGTGCTCGCGCTCCT
<<< < << <<< <<<<<< <<< < <

601 CGACGGCGAGGGCCGCGTCGTGGACGTGCTCGAGGACCGGGGCCGcGAGGTGATGAAGCT

661 GGTGAGCGAGGTGCGGGAGGTGGGCGGCAAGCTGTGGATCGGGACAGTGGCGCACAACCA

721 CATCGCCACGGTGCCCTACCCCTTATTGGACTAGCCACAGCGTGCCATTTCCATTGTTAC

781 TACTGGCTCCTAGTATTATTATGTGTACTACGTCTATAATGGAGTGTGCGATGACTGGTC

841 GTGTTCTTGATTGTACGTATTTGGCATAACAATGGACAAACTTCAGATCAGAAATCGAAT

901 AAAATTACTAGTCATGGTTGCT

# i23387

Homolog to four isotigs in the sexual database (SEXisotig04501, SEXisotig04502, SEXisotig04503 and SEXisotig04504) and two in the apomictic one (APOisotig06353 and APOisotig06354)

>SEXisotig04501 gene=isogroup00780 length=1214 numContigs=4
CTCGGCTCTCAAGTGCCACTTGTTCTCCCtCCTCTTGTCTGCCTCAGGCCGGATCAGGCG
ATCAGCGCAGCGTGAAGCAGCAGCAGCAGCAATGGCGATGGTGTCGTCGAGTGGGCTGAG
GAGCTGCAGCGCCGTGGGCGTGCCTCGCAGCCTACGGGCATCGTCGTCCTCGTCCGGCCG
CCTGCCGGTCTGCGCTAACGCCACCACATCCGGCCGCGTTACCATGTCCGCCGAGTGGAT

GCCCGGCCAGCCCCGCCCCGCCCACCTCGATGGCTCCTCTCCCGGGGACTTCGGCTTCGA
CCCCCTTGGCCTCGCCACCGTGCCGGAGAACTTCGAGCGGTTCAAGGAGTCCGAGGTCTA
CCACTGCCGCTGGGCCATGCTCGCTGTGCCCGGGATCCTGGTGCCGGAGGCGCTGGGTCT
TGGCAACTGGGTGAAGGCGCAGGAGTGGGCGGCCGTCCCCGGCGGTCAGGCGACGTACCT
CGGCAACCCGGTGCCATGGGGCTCCCTGCCCACCATCCTGGCGATCGAGTTCGTGGCCAT
CGCCTTCGCCGAACACCAGCGCACCATGGAGAAGGACCCCGAGAAGAAGAAATACCCCGG
CGGTGCCTTTGACCCGCTCGGCTTCTCCAAGGACCCCGTCAAGTTCGAGGAATACAAGCT
CAAGGAGATCAAGAACGGTATGTCCCGCTGCAATTCTTCAGCGCCCTTTtCGCTGCATCC
TTCATGCAATTGCACCGCGAATACTTAATTAGCTAACCAACCAACCATTGTCTGAACTGA
ATGTGTGCAGGCCGGCTGGCGATGTTGGCGTTCGTTGGTTTCTGCGTGCAGCAGTCGGCG
TACCCTGGCACCGGGCCACTGGAGAACCTGGCCACCCACCTTGCCGACCCATGGCACAAC
AACATCGGCGACATTATCATCCCCAGAACAATCTACCCTTGAGCTGAGATGATTGCATTT
GCGTGGACGAATATGAAATGAAACGAGCTGTGCTGGGAGTGTGGTCGGTGTTTTTGTGTG
TGTGATTGGTTGATTACTAGGCACTGCCTGCGTGTGTGCAGGAACTGTACAACGACGACG
GCGACTTCCTGGCCGGAAGCCCCATGTGATGTAAAGCTTATTATTAACTTTTGCATCAAA
TATAAACTGGTTAGTAGCTAGcATCAGCATCTCTtAaCTATGAACTAGTaTTAATCAATA
GCTCATATATACAA

Putative intron
LEFT PRIMER: CTTTtCGCTGCATCCTTCAT
RIGHT PRIMER: CGTCGTCGTTGTACAGTTCC

**Primers designed on internal sequences of the putative intron**

---

PRIMER PICKING RESULTS FOR SEXisotig04501 gene=isogroup00780 length=1214 numContigs=4

No mispriming library specified
Using 1-based sequence positions

| OLIGO | start | len | tm | gc% | any | 3' | seq |
|---|---|---|---|---|---|---|---|
| LEFT PRIMER | 706 | 20 | 60.35 | 45.00 | 4.00 | 2.00 | CTTTtCGCTGCATCCTTCAT |
| RIGHT PRIMER | 1080 | 20 | 59.21 | 55.00 | 6.00 | 1.00 | CGTCGTCGTTGTACAGTTCC |

SEQUENCE SIZE: 1214
INCLUDED REGION SIZE: 1214

PRODUCT SIZE: 375, PAIR ANY COMPL: 3.00, PAIR 3' COMPL: 0.00
TARGETS (start, len)*: 751,270

1 CTCGGCTCTCAAGTGCCACTTGTTCTCCCtCCTCTTGTCTGCCTCAGGCCGGATCAGGCG

61 ATCAGCGCAGCGTGAAGCAGCAGCAGCAGCAATGGCGATGGTGTCGTCGAGTGGGCTGAG

121 GAGCTGCAGCGCCGTGGGCGTGCCTCGCAGCCTACGGGCATCGTCGTCCTCGTCCGGCCG

181 CCTGCCGGTCTGCGCTAACGCCACCACATCCGGCCGCGTTACCATGTCCGCCGAGTGGAT

241 GCCCGGCCAGCCCCGCCCCGCCCACCTCGATGGCTCCTCTCCCGGGGACTTCGGCTTCGA

301 CCCCCTTGGCCTCGCCACCGTGCCGGAGAACTTCGAGCGGTTCAAGGAGTCCGAGGTCTA

361 CCACTGCCGCTGGGCCATGCTCGCTGTGCCCGGGATCCTGGTGCCGGAGGCGCTGGGTCT

421 TGGCAACTGGGTGAAGGCGCAGGAGTGGGCGGCCGTCCCCGGCGGTCAGGCGACGTACCT

481 CGGCAACCCGGTGCCATGGGGCTCCCTGCCCACCATCCTGGCGATCGAGTTCGTGGCCAT

541 CGCCTTCGCCGAACACCAGCGCACCATGGAGAAGGACCCCGAGAAGAAGAAATACCCCGG

601 CGGTGCCTTTGACCCGCTCGGCTTCTCCAAGGACCCCGTCAAGTTCGAGGAATACAAGCT

661 CAAGGAGATCAAGAACGGTATGTCCCGCTGCAATTCTTCAGCGCCCTTTtCGCTGCATCC
>>>>>>>>> >> >>>>

```
721 TTCATGCAATTGCACCGCGAATACTTAATTAGCTAACCAACCAACCATTGTCTGAACTGA
    >>>>> *******************************************************

781 ATGTGTGCAGGCCGGCTGGCGATGTTGGCGTTCGTTGGTTTCTGCGTGCAGCAGTCGGCG
    ************************************************************

841 TACCCTGGCACCGGGCCACTGGAGAACCTGGCCACCCACCTTGCCGACCCATGGCACAAC
    ************************************************************

901 AACATCGGCGACATTATCATCCCCAGAACAATCTACCCTTGAGCTGAGATGATTGCATTT
    ************************************************************

961 GCGTGGACGAATATGAAATGAAACGAGCTGTGCTGGGAGTGTGGTCGGTGTTTTTGTGTG
    ************************************************************

1021 TGTGATTGGTTGATTACTAGGCACTGCCTGCGTGTGTGCAGGAACTGTACAACGACGACG
                                             < <<<  <<< < <<< <<<<< <<< <

1081 GCGACTTCCTGGCCGGAAGCCCCATGTGATGTAAAGCTTATTATTAACTTTTGCATCAAA

1141 TATAAACTGGTTAGTAGCTAGcATCAGCATCTCTtAaCTATGAACTAGTaTTAATCAATA

1201 GCTCATATATACAA
```

## i11548

Homolog to one isotig in the sexual database (SEXisotig22583) and three in the apomictic one (APOisotig02915, APOisotig02916 and APOisotig02917)

```
>APOisotig02916  gene=isogroup00353  length=1266  numContigs=5
ttttGGAACcTTAGACgTGAGATCAATTACTTAAGCACAAACTGCAAACTCAGACAGCAT
TCGTATCAGAGATCAGACCGAAAAGGTTTTCTTGCCCTCTTCATAATTTACACGCATGGC
ACATTCATCAGACCCTTACACAAACATTTTTCCCGGTAAAATCAAACACGGCAGCACAGC
ATGTGAAAGGCCATCGCCTGGACGTCCTCACTCACAGGCTGGGCGTCCTCTCGGCGGCGC
CAGAGATGACGGTGAGCAGGTTGTTGCCGAAGGGGTCACTGAGGTGCTTGGCGAGGTTCT
CGACGGGGCCTTCGCCGGTGACGTAGGCCTGGATGAAGAAGGCGAACATGGAGAACATGG
CCAGCCGGCCGTTCTTGATCTCCTTCACCTTGAGgAGCGCGGCCTGGTCGGGGTCGCTCG
CCAGGCCCAGGGGGTCGAAGGGGCCTCCCGGGTGCAGCTTGTCCTCAAGGTCCAGGCCGT
TGGTGATCCGGTAGTACTCGGCGCCGCCGACCAGGACGATCTCGGCGATCACCGCCACCA
CCAGGTTGATGGGGATGCTGTTGCCGAAGTAGTTGAGGGTGTTGCCGTCCAGGAGCAGGG
CGCCCGTCTGCATTGCATTGATTGAGGTGAGCACCATACATGCATGAGTTtCAGACATTC
AGGTTCAGATATTTGAGAAAGAAAGCAAAAAaTTGCTTCTGAAGCATGAGGAAGGTGATC
ATCAGGACCTTGAACCAGACGGCCTCAGGGCCACAGTTGGCGCCGAACTTGTTGCACGCC
TCAGGGATGACGGCACCGGCAGCGCCGAGCATGGCCCACCTGGCATGGATCAGCTCATAG
GCCTGGTACTTGGCGAAATCCTCTGGCTTCTTGCCAAGGCCAAATGGGTCGTAGCCATAG
TCTCCTGGGACCTCTCCGGTGAGGTACTCCGGCACCTCCGAGCGGTCCAAGAGACCATCC
GGCAGGTAGATCCTCCTGTCAGGGCCATACCACTTGGCGAGCTCGTCGCTGATGTCCGGG
GACGAGGACGATACCGCCGCAGGCTTTGGCTTCTGGGCAGGCTTCTTGCTGAAGAGGGAG
ACGATCTTCTGCGCCCCAGCGGTCGGTGCCGCCGTGGCGTAGCGAGAAGACCCAGCGAAG
TTTAGCTGGGTTCCGAGGATCTTCGAAGGAGCGAGCGCCGCCATTGTCGCCGACCCACAG
CTCAGAGGGAAGATTCTCAGCAGGAGGGGAGGCTGAGAGACggagtggtcggcgtcttca
agggca
```

Putative intron 1
LEFT PRIMER: GAGGTGAGCACCATACATGC
RIGHT PRIMER: CAAGAAGCCAGAGGATTTCG

**Primers designed on internal sequences of the putative intron**

---

PRIMER PICKING RESULTS FOR APOisotig02916  gene=isogroup00353  length=1266  numContigs=5

No mispriming library specified
Using 1-based sequence positions

| OLIGO | start | len | tm | gc% | any | 3' | seq |
|---|---|---|---|---|---|---|---|
| LEFT PRIMER | 624 | 20 | 59.12 | 55.00 | 6.00 | 2.00 | GAGGTGAGCACCATACATGC |
| RIGHT PRIMER | 873 | 20 | 59.95 | 50.00 | 3.00 | 2.00 | CAAGAAGCCAGAGGATTTCG |

SEQUENCE SIZE: 1266
INCLUDED REGION SIZE: 1266

PRODUCT SIZE: 250, PAIR ANY COMPL: 2.00, PAIR 3' COMPL: 1.00
TARGETS (start, len)*: 720,121

1 ttttGGAACcTTAGACgTGAGATCAATTACTTAAGCACAAACTGCAAACTCAGACAGCAT

61 TCGTATCAGAGATCAGACCGAAAAGGTTTTCTTGCCCTCTTCATAATTTACACGCATGGC

121 ACATTCATCAGACCCTTACACAAACATTTTTCCCGGTAAAATCAAACACGGCAGCACAGC

181 ATGTGAAAGGCCATCGCCTGGACGTCCTCACTCACAGGCTGGGCGTCCTCTCGGCGGCGC

241 CAGAGATGACGGTGAGCAGGTTGTTGCCGAAGGGGTCACTGAGGTGCTTGGCGAGGTTCT

301 CGACGGGGCCTTCGCCGGTGACGTAGGCCTGGATGAAGAAGGCGAACATGGAGAACATGG

361 CCAGCCGGCCGTTCTTGATCTCCTTCACCTTGAGgAGCGCGGCCTGGTCGGGGTCGCTCG

421 CCAGGCCCAGGGGGTCGAAGGGGCCTCCCGGGTGCAGCTTGTCCTCAAGGTCCAGGCCGT

481 TGGTGATCCGGTAGTACTCGGCGCCGCCGACCAGGACGATCTCGGCGATCACCGCCACCA

541 CCAGGTTGATGGGGATGCTGTTGCCGAAGTAGTTGAGGGTGTTGCCGTCCAGGAGCAGGG

601 CGCCCGTCTGCATTGCATTGATTGAGGTGAGCACCATACATGCATGAGTTtCAGACATTC
>> >>> >> >>>> >> >>>> >>>

661 AGGTTCAGATATTTGAGAAAGAAAGCAAAAAaTTGCTTCTGAAGCATGAGGAAGGTGATC
*

721 ATCAGGACCTTGAACCAGACGGCCTCAGGGCCACAGTTGGCGCCGAACTTGTTGCACGCC
*********************************************************************************************************

781 TCAGGGATGACGGCACCGGCAGCGCCGAGCATGGCCCACCTGGCATGGATCAGCTCATAG
************************************************************

841 GCCTGGTACTTGGCGAAATCCTCTGGCTTCTTGCCAAGGCCAAATGGGTCGTAGCCATAG
<<< <<<< <<<< <<< <<<<<<

901 TCTCCTGGGACCTCTCCGGTGAGGTACTCCGGCACCTCCGAGCGGTCCAAGAGACCATCC

961 GGCAGGTAGATCCTCCTGTCAGGGCCATACCACTTGGCGAGCTCGTCGCTGATGTCCGGG

1021 GACGAGGACGATACCGCCGCAGGCTTTGGCTTCTGGGCAGGCTTCTTGCTGAAGAGGGAG

1081 ACGATCTTCTGCGCCCCAGCGGTCGGTGCCGCCGTGGCGTAGCGAGAAGACCCAGCGAAG

1141 TTTAGCTGGGTTCCGAGGATCTTCGAAGGAGCGAGCGCCGCCATTGTCGCCGACCCACAG

1201 CTCAGAGGGAAGATTCTCAGCAGGAGGGGAGGCTGAGAGACggagtggtcggcgtcttca

1261 agggca

KEYS (in order of precedence):
****** target
>>>>>> left primer
<<<<<< right primer

# i22343

Homolog to two isotigs in the sexual database (SEXisotig10057 and SEXisotig10058) and one in the apomictic one (APOisotig24343)

>SEXisotig10057  gene=isogroup03264  length=1346  numContigs=3
GAAACTTTGATTTcCAGCAAtTTTGtTGCCAAATTTGAtTTTTcTTTCACGTAACTGGATCACA
ATTCTTCACAACTATACGTTAGGATACATAGAATTTACTGACTTATATATCTTTTCCTGTAA
AGAGCATGCCCTTACATGACCTGGTGTATGACACCAGCTAATCTCTGAAAGTGCACACAC
CCACAGCCCACTTCTGGTAACTTGAACCAATCATATATATGTATGTATCAACTAAAtGtATG
GAAGTATGTAAatGCTAGAGAGGTAGCTGCTaATAAGCTTGATCTTGcATATGTATATGAAC
TATATATATATGATGCTGGCTGAATCATTCATGGCCTCTCTGACGAGTTCCGTGCTCCGAT
TGAAATGGCTAAGGTGACGAGGCTGCATGGCACAGAACAGGCATAATCAGACATCTACT
TCAAGAGCAATATGTGAAACATTAACTAAAGAACAAGACAATATTATCTTGAAGAAGAATT
GTACTAATAGAGTGTATTATTGCTTGCCTGAAGTCGGCTTCTCCCAGACgTCAGGAATGC
GGCCCACGAGCGGCGGGGACTGCTGCCCAGTGAAGCAGATGTAGAGGACTTGTGGCG
GCAGGACGACGCCACAAGCAATGGGCAGGTACATCATGCGCACGGGATCCACAGGCTT
GGGCAGCATCTTGTTCACGTCCCCGAGGATCACCGGGCACAGGCACCGCAGCGTGCGG
TCGTCGCCGGGCGCCGCCGCGCTGGAGCTGTTGAGGAAGGCGCCGAGGCCGTTGCAG
CACTCGCTCTGGGGCGTCGGCGTGTCCGGCTCGCTCCCCGTCAGGAACGCCCCGCAGG
TCATCAGCCCCGCGAGCGGTGTCACGCACTCCGTCGGCGACGACGGCGTGGAGCCGCC
GACGCTGCCCGGCAGGCCGGGGAAGCCCGGCAGCTGCGGCACCGCCGGCCTTGAGCA
AGGCGGCGGCGACCCGGGCGCCGACGACGGCACGCCGCCGGGCGTGGGCGCCGGTA
CACCTGGGACGACGGACCCGGACGTCGGCGGCGACGACGTCCTGCCTCTGGGGTAGCC
CGGGAACACGGGGTTGCGCGGCCGGCTGCCCGGGCGGCCGGGGAACCGGAGCGCGT
CGGCGTCCGGCGGCGGGTTGCTCGCCGCCGATGGCTGCACCGTCATGAGAACGGCAG
CGACGGCGATCACTACGGCGGCAGCGTGCTTCTTGGCCATGGATGCTGCTTTTCTTGCT
ACTCGATCACTTGCCTGTGGATTGCTGTGACAGATTCAAACACGAGAGCAGGAGGAGAT
CAAATGCAGCgGTCTGTGGATGGcTGTGaCGaTcCATTGccCCCGCC

Putative intron
LEFT PRIMER: CATGGCACAGAACAGGCATA
RIGHT PRIMER: CTTGTTCACGTCCCCGAG

**Primers designed on internal sequences of the putative intron**

---

PRIMER PICKING RESULTS FOR SEXisotig10057  gene=isogroup03264  length=1346  numContigs=3

No mispriming library specified
Using 1-based sequence positions

| OLIGO | start | len | tm | gc% | any | 3' | seq |
|---|---|---|---|---|---|---|---|
| LEFT PRIMER | 397 | 20 | 60.69 | 50.00 | 4.00 | 2.00 | CATGGCACAGAACAGGCATA |
| RIGHT PRIMER | 693 | 18 | 60.24 | 61.11 | 4.00 | 2.00 | CTCGGGGACGTGAACAAG |

SEQUENCE SIZE: 1346
INCLUDED REGION SIZE: 1346

PRODUCT SIZE: 297, PAIR ANY COMPL: 3.00, PAIR 3' COMPL: 0.00
TARGETS (start, len)*: 421,251

1 GAAACTTTGATTTcCAGCAAtTTTGtTGCCAAATTTGAtTTTTcTTTCACGTAACTGGAT

61 CACAATTCTTCACAACTATACGTTAGGATACATAGAATTTACTGACTTATATATCTTTTC

121 CTGTAAAGAGCATGCCCTTACATGACCTGGTGTATGACACCAGCTAATCTCTGAAAGTGC

```
181 ACACACCCACAGCCCACTTCTGGTAACTTGAACCAATCATATATATGTATGTATCAACTA

241 AAtGtATGGAAGTATGTAAatGCTAGAGAGGTAGCTGCTaATAAGCTTGATCTTGcATAT

301 GTATATGAACTATATATATATGATGCTGGCTGAATCATTCATGGCCTCTCTGACGAGTTC

361 CGTGCTCCGATTGAAATGGCTAAGGTGACGAGGCTGCATGGCACAGAACAGGCATAATCA
                                    >>>>>>>>>>>>>>>>>>>>

421 GACATCTACTTCAAGAGCAATATGTGAAACATTAACTAAAGAACAAGACAATATTATCTT
    ************************************************************

481 GAAGAAGAATTGTACTAATAGAGTGTATTATTGCTTGCCTGAAGTCGGCTTCTCCCAGAC
    ************************************************************

541 gTCAGGAATGCGGCCCACGAGCGGCGGGGACTGCTGCCCAGTGAAGCAGATGTAGAGGAC
    ************************************************************

601 TTGTGGCGGCAGGACGACGCCACAAGCAATGGGCAGGTACATCATGCGCACGGGATCCAC
    ************************************************************

661 AGGCTTGGGCAGCATCTTGTTCACGTCCCCGAGGATCACCGGGCACAGGCACCGCAGCGT
    ***********    <<<<<<<<<<<<<<<<<<<

721 GCGGTCGTCGCCGGGCGCCGCCGCGCTGGAGCTGTTGAGGAAGGCGCCGAGGCCGTTGCA

781 GCACTCGCTCTGGGGCGTCGGCGTGTCCGGCTCGCTCCCCGTCAGGAACGCCCCGCAGGT

841 CATCAGCCCCGCGAGCGGTGTCACGCACTCCGTCGGCGACGACGGCGTGGAGCCGCCGAC

901 GCTGCCCGGCAGGCCGGGGAAGCCCGGCAGCTGCGGCACCGCCGGCCTTGAGCAAGGCGG

961 CGGCGACCCGGGCGCCGACGACGGCACGCCGCCGGGCGTGGGCGCCGGTACACCTGGGAC

1021 GACGGACCCGGACGTCGGCGGCGACGACGTCCTGCCTCTGGGGTAGCCCGGGAACACGGG

1081 GTTGCGCGGCCGGCTGCCCGGGCGGCCGGGGAACCGGAGCGCGTCGGCGTCCGGCGGCGG

1141 GTTGCTCGCCGCCGATGGCTGCACCGTCATGAGAACGGCAGCGACGGCGATCACTACGGC

1201 GGCAGCGTGCTTCTTGGCCATGGATGCTGCTTTTCTTGCTACTCGATCACTTGCCTGTGG

1261 ATTGCTGTGACAGATTCAAACACGAGAGCAGGAGGAGATCAAATGCAGCgGTCTGTGGAT

1321 GGcTGTGaCGaTcCATTGccCCCGCC
```

KEYS (in order of precedence):
****** target
>>>>>> left primer
<<<<<< right primer

## **i24572**

Homolog to one isotig in sexual database (SEXisotig25401) and two in the apomictic one (APOisotig14809 and APOisotig14810)

>isotigAPO14809 gene=isogroup05204 length=889 numContigs=3
ACAgTACACACAGGTCCAAaCTCACAgTACAGGTGGGAACAGTCCCATCTCACAGTCACT
CGATCACACACGACCAAGGAAGGGTAGGGACATTTtATTCCTGGgTTGggTGTTTTtCAT
CTTATTATGGATCGATCTCAAGAACACCACCGTGACGACGACACACAGGGACAGGGGTAG
CGCGCCTAGTGCTGTCCAGGCAGCTTCTCCTTGATCTTGTCCATGATGCCCTTCTTCTCG
CCGGTGCCGTCGGTGGTGCCATAAGTAGTAGTCCCGGTGCCGGTGTGTCCCTGCGGATGC
CCGTAGGCGGCGCCGGTGGCGGTGGCATGCTGCTGGTCTGCTGCTTCCTGCTTGTGGCCA
GCTCCGGGCATTTTCTCCTTGATCTTCTCCTTGAGGCCCTTCTTCCTCCTCCCGCCCATG
CCGTCGTCCTCAGACTACACATATACCATGCgTCACAAAGACATGCCTTAATTATATTAC
AAATcacAAGCGAGGGgACGGAgaaGTACATATAAGTATGCCACAAtaaTAAGGAAatag
TAATAAcgtaCGTACCGAGCTGGAGCTGGAGCTGCCGGAGCGGTGCAGGACACCGCCGGT
CTTGTGCTCCTCCCTCACCGGCTGGAACTGCCCGCCGCCCATGCCGTGCTGGCCCGTCAT
GGCTCCTCCGGTTCCGGTCCCCATGCCAGCTCCACCGGCGGTGGTTCCGACCGGGTTGCC
GTACTGGTCGACGCGGCCGGTCCCGGTGGTGGTGCCGTGGCCGTGCTGCTGACCGTACTC
CATGCTAGCTACTGGTTGGCTTGGTTCTTCCTCTCCCTTTAAAGCTAGGTAGTAGCGAGA
TGAGCTAGTGCTGTGTGGTTGTGTTCTCTGCTCGgCTCACtGATTTGTT

Putative intron 1
LEFT PRIMER: ACCATGCgTCACAAAGACAT
RIGHT PRIMER: GTCGACCAGTACGGCAACC

**Primers designed on internal sequences of the putative intron**

---

PRIMER PICKING RESULTS FOR isotigAPO14809 gene=isogroup05204 length=889 numContigs=3

No mispriming library specified
Using 1-based sequence positions
OLIGO start len tm gc% any 3' seq
LEFT PRIMER 445 20 59.01 45.00 4.00 2.00 ACCATGCgTCACAAAGACAT
RIGHT PRIMER 732 19 61.54 63.16 6.00 2.00 GTCGACCAGTACGGCAACC
SEQUENCE SIZE: 889
INCLUDED REGION SIZE: 889

PRODUCT SIZE: 288, PAIR ANY COMPL: 3.00, PAIR 3' COMPL: 0.00
TARGETS (start, len)*: 500,175

```
  1 ACAgTACACACAGGTCCAAaCTCACAgTACAGGTGGGAACAGTCCCATCTCACAGTCACT


 61 CGATCACACACGACCAAGGAAGGGTAGGGACATTTtATTCCTGGgTTGggTGTTTTtCAT


121 CTTATTATGGATCGATCTCAAGAACACCACCGTGACGACGACACACAGGGACAGGGGTAG


181 CGCGCCTAGTGCTGTCCAGGCAGCTTCTCCTTGATCTTGTCCATGATGCCCTTCTTCTCG


241 CCGGTGCCGTCGGTGGTGCCATAAGTAGTAGTCCCGGTGCCGGTGTGTCCCTGCGGATGC


301 CCGTAGGCGGCGCCGGTGGCGGTGGCATGCTGCTGGTCTGCTGCTTCCTGCTTGTGGCCA


361 GCTCCGGGCATTTTCTCCTTGATCTTCTCCTTGAGGCCCTTCTTCCTCCTCCCGCCCATG


421 CCGTCGTCCTCAGACTACACATATACCATGCgTCACAAAGACATGCCTTAATTATATTAC
                            >>>> >>>>>> >>>> >> >>> >

481 AAATcacAAGCGAGGGgACGGAgaaGTACATATAAGTATGCCACAAtaaTAAGGAAatag
    ************************************************************

541 TAATAAcgtaCGTACCGAGCTGGAGCTGGAGCTGCCGGAGCGGTGCAGGACACCGCCGGT
    ************************************************************

601 CTTGTGCTCCTCCCTCACCGGCTGGAACTGCCCGCCGCCCATGCCGTGCTGGCCCGTCAT
    ************************************************************

661 GGCTCCTCCGGTTCCGGTCCCCATGCCAGCTCCACCGGCGGTGGTTCCGACCGGGTTGCC
```

```
      **************                                                            <<<<<<<

721 GTACTGGTCGACGCGGCCGGTCCCGGTGGTGGTGCCGTGGCCGTGCTGCTGACCGTACTC
    <<< <<< <<< <<<

781 CATGCTAGCTACTGGTTGGCTTGGTTCTTCCTCTCCCTTTAAAGCTAGGTAGTAGCGAGA


841 TGAGCTAGTGCTGTGTGGTTGTGTTCTCTGCTCGgCTCACtGATTTGTT
```

KEYS (in order of precedence):
****** target
>>>>>> left primer
<<<<<< right primer
