# Supplementary material for: Spotting the Targets of the Apospory Controller TGS1 in Paspalum notatum
Source: Plants (Basel). 2022 Jul 26;11(15):1929. doi: 10.3390/plants11151929 (PMC9332697; doi:10.3390/plants11151929)
Supplement: Supplementary file 1 [file plants-11-01929-s001.zip › Supplementary Figure S1.pdf]

Supplementary Figure S1

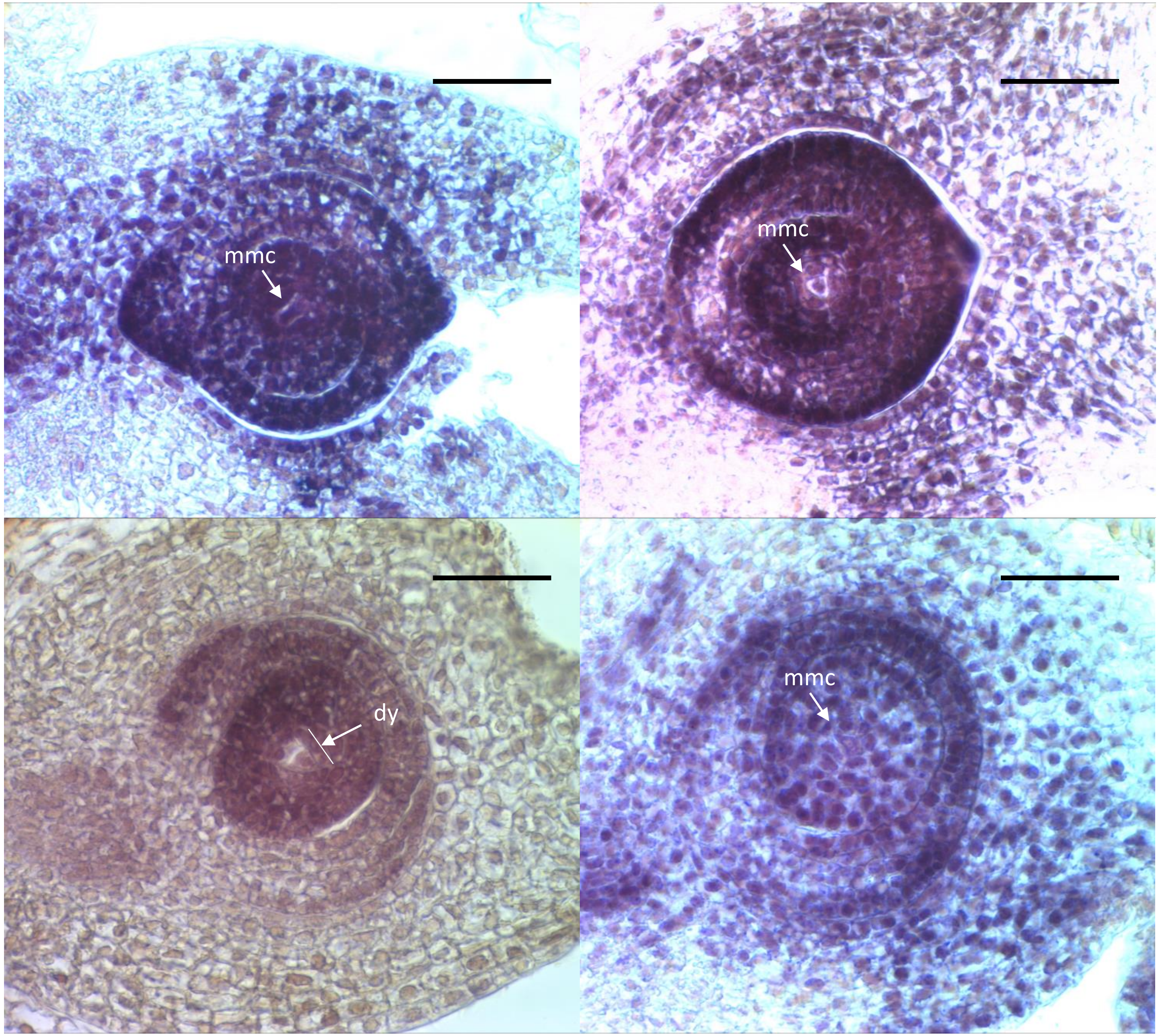

RNA *in situ* hybridization of premeiotic/meiotic ovules with *QGJ* antisense probe. Plant material: sexual genotype Q4188. dy: dyad; mmc: megaspore mother cell. Bars: 20 µm.

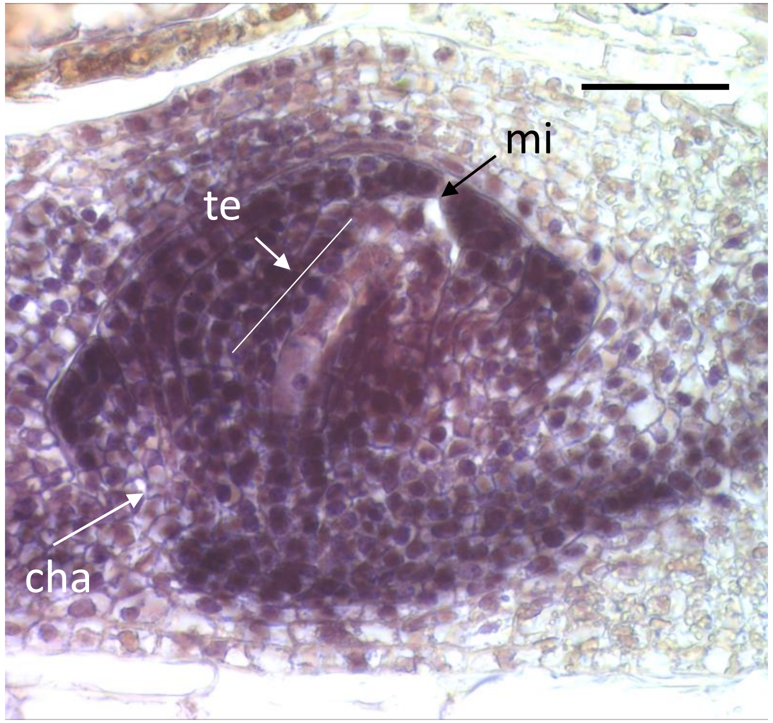

RNA *in situ* hybridization of meiotic ovules with the *QGJ* antisense probe. Plant material: sexual genotype Q4188. cha: chalaza; te: tetrad. Bars: 20 µm.

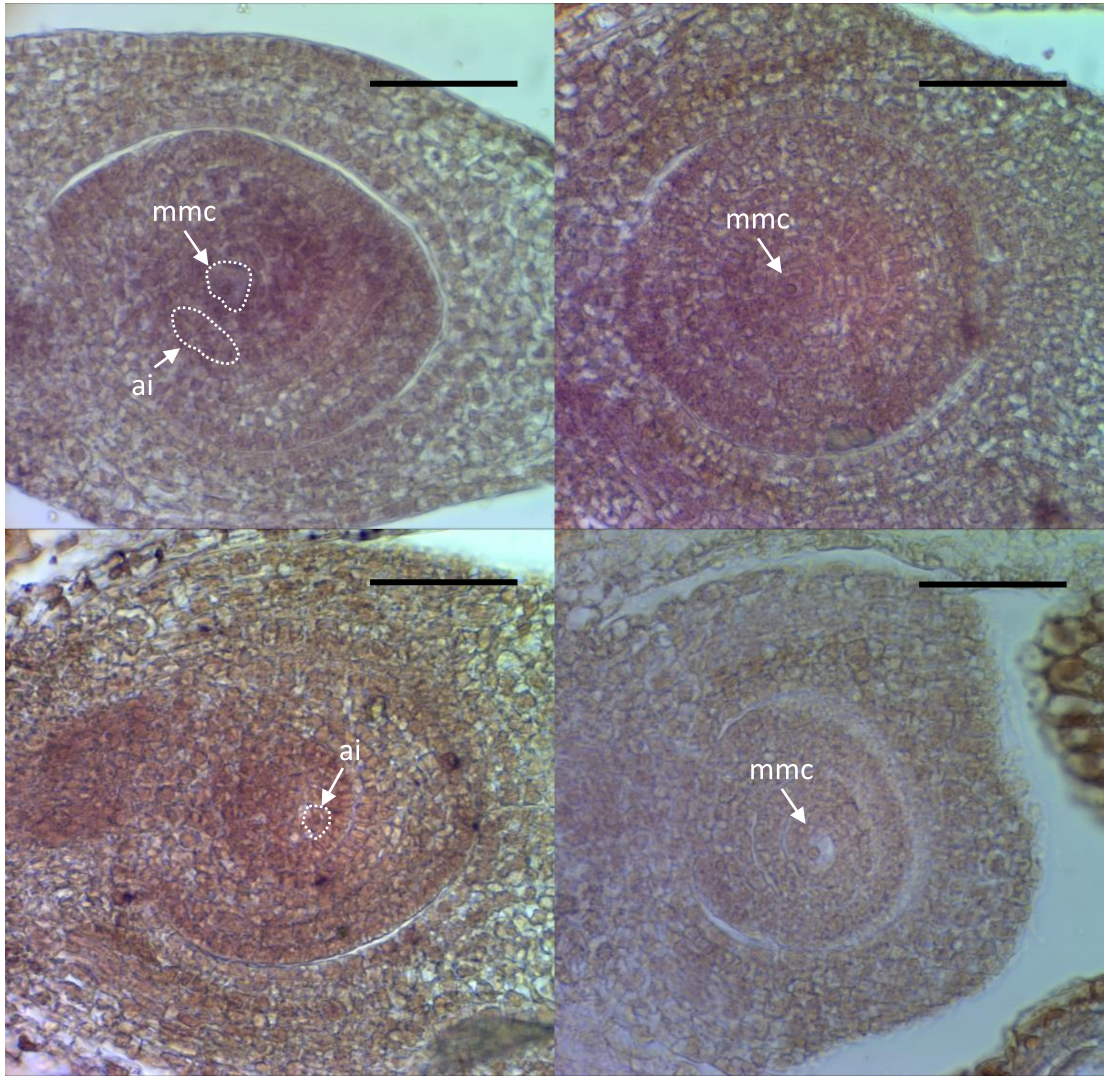

RNA *in situ* hybridization of premeiotic ovules with the *QGJ* antisense probe. Plant material: sexual *tgs1* defective line. ai: putative apospory initial; mmc: megaspore mother cell. Bars: 20 µm.

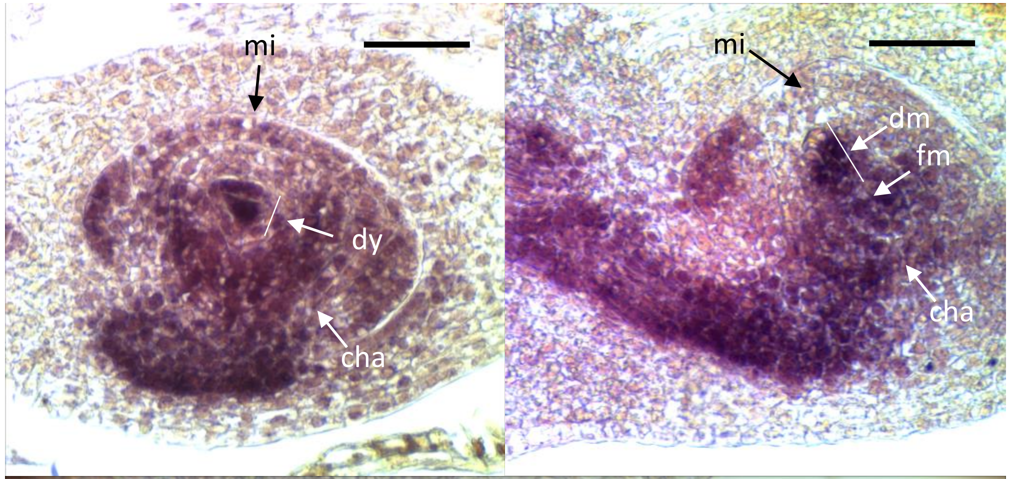

RNA *in situ* hybridization of meiosis-stage ovules with the *QGJ* antisense probe. Plant material: *tgs1* defective line. cha: chalaza; dm: degenerating megaspores; dy: dyad; fm: functional megaspores; mi: micropyle. Bars: 20 μm.

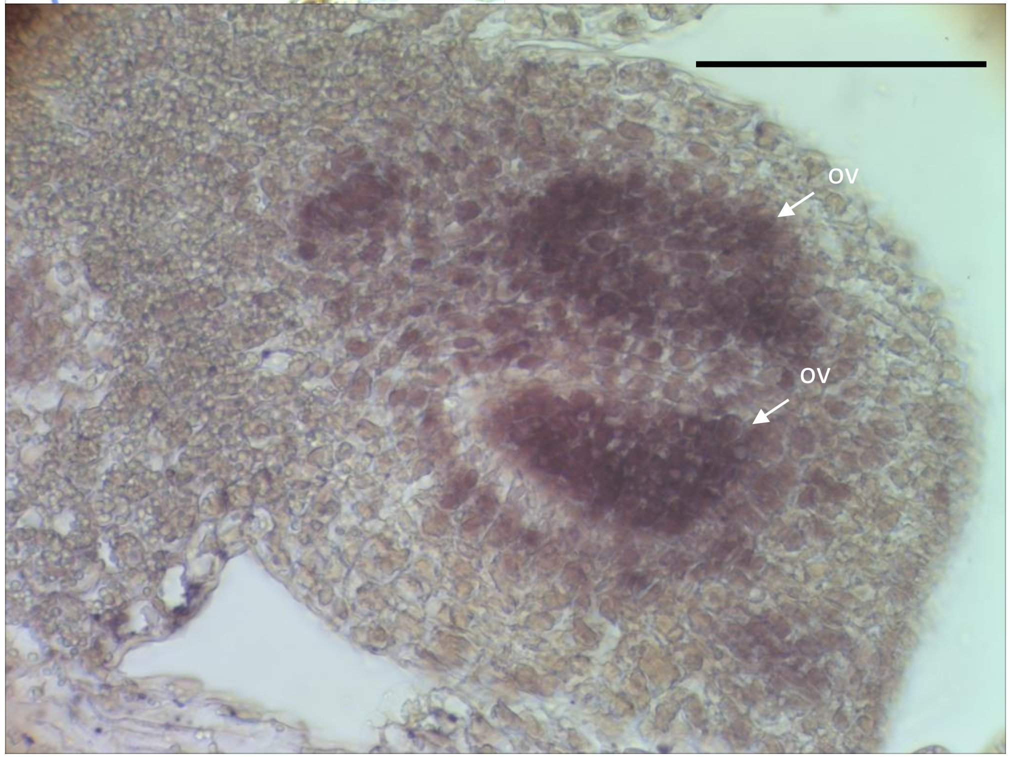

RNA *in situ* hybridization of twin ovules with the *QGJ* antisense probe. Plant material: sexual *tgs1* defective line. ov: ovule. Bars: 20 μm.
