# Supplementary material for: Spotting the Targets of the Apospory Controller TGS1 in Paspalum notatum
Source: Plants (Basel). 2022 Jul 26;11(15):1929. doi: 10.3390/plants11151929 (PMC9332697; doi:10.3390/plants11151929)

Supplementary Figure S2

Stem-loop RT-PCR scheme for miRNA2275a (A) and miRNA168 (B). 1: Reverse transcription of miRNA primed by specific stem-loop RT primer. 2: First-strand cDNAs are amplified by using specific forward primer and reverse primer complementary to the stem-loop RT primer. The purpose of the tailed forward primer is to increase ihe melting temperature (Tm) depending on the sequence composition of miRNA molecules. The graphic was produced using sRNAPrimerDB (http://www.srnaprimerdb.com.).

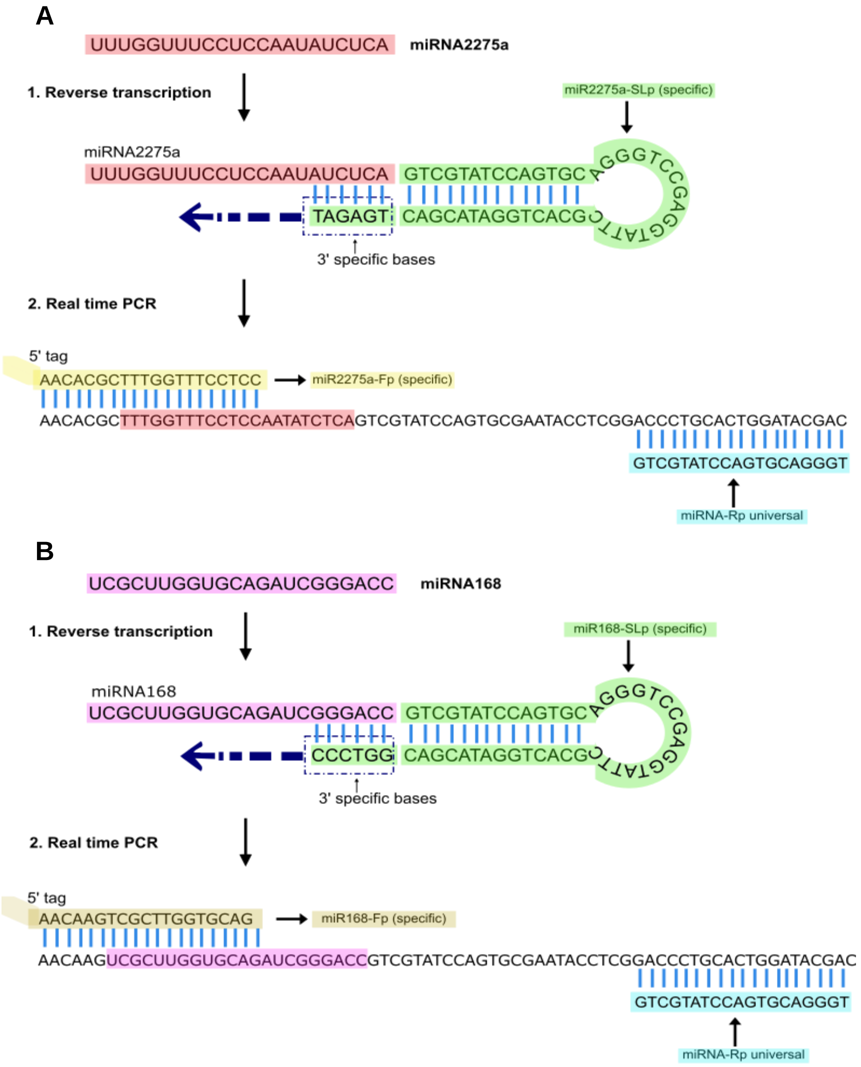

Supplement: Supplementary file 1 [file plants-11-01929-s001.zip › Supplementary Figure S2.pdf]
