# Supplementary material for: Spotting the Targets of the Apospory Controller TGS1 in Paspalum notatum
Source: Plants (Basel). 2022 Jul 26;11(15):1929. doi: 10.3390/plants11151929 (PMC9332697; doi:10.3390/plants11151929)
Supplement: Supplementary file 1 [file plants-11-01929-s001.zip › Supplementary Table S1.pdf]

Supplementary Table S1: Twenty (20) selected transcripts representing putative differentially-expressed splice variants in libraries of apomictic and sexual *P. notatum* plants [34].

Transcript	Reads Apo	Reads Sex	Annotation	p-value for differential expression	FDR for differential expression
**i17272**	243	0	Full=Non-specific lipid-transfer protein 2B	1.55E-73	1.03E-69
**i01046**	206	0	Full=Non-specific lipid-transfer protein 2	1.41E-62	7.69E-59
**i39328**	233	5	.	2.70E-62	1.35E-58
**i15773**	2	171	.	1.95E-47	4.04E-44
**i01798**	139	0	Full=Auxin-repressed 12.5 kDa protein	2.33E-42	3.99E-39
**i10779**	139	458	Full=Tetraketide alpha-pyrone reductase 1	8.99E-38	1.28E-34
**i17045**	104	0	.	6.47E-32	5.97E-29
**i47485**	96	327	.	8.94E-29	6.79E-26
**i17370**	191	467	Full=Stem-specific protein TSJT1	2.18E-25	1.34E-22
**i05665**	84	0	Full=17.5 kDa class II heat shock protein	6.17E-26	3.94E-23
**i07995**	80	0	.	9.72E-25	5.72E-22
**i38131**	79	0	Full=60 kDa jasmonate-induced protein	1.94E-24	1.13E-21
**i18863**	28	166	.	7.13E-24	3.99E-21
**i08637**	77	0	Full=Cytochrome P450 76C3	7.68E-24	4.27E-21
**i07994**	76	0	.	1.53E-23	8.43E-21
**i22630**	144	377	Full=Strictosidine synthase	2.21E-23	1.18E-20
**i23387**	490	235	Full=Chlorophyll a-b binding protein 1B-21	2.98E-22	1.50E-19
**i11548**	729	411	Full=Chlorophyll a-b binding protein CP26	8.11E-22	3.99E-19
**i22343**	164	397	.	1.84E-21	8.95E-19
**i24572**	18	105	Full=Dehydrin DHN1	8.14E-16	2.43E-13
