# Supplementary material for: Spotting the Targets of the Apospory Controller TGS1 in Paspalum notatum
Source: Plants (Basel). 2022 Jul 26;11(15):1929. doi: 10.3390/plants11151929 (PMC9332697; doi:10.3390/plants11151929)
Supplement: Supplementary file 1 [file plants-11-01929-s001.zip › Supplementary Table S2.pdf]

Supplementary Table S2. Primers used in PCR, qPCR and stem-loop qPCR analysis

Amplicon	Name of primer	Sequence (5′-3′)	Length (bp)	Product size (bp)	Tm (°C)
i10779	i10779 upper	5′-CTCGCATGATAGGCTGAGTG-3′	20	244	59.57
	i10779 lower	5′-ATTGGCAGAGAAAGCAGCAT-3′	20		59.98
i22630	i22630 upper	5′-CAGAAGATTATAGAGCAGCCAATG-3′	24	291	59.46
	i22630 lower	5′-GCTCTCCTCGTGAGCATCTT-3′	20		59.71
i23387	i23387 upper	5′-CTTTTCGCTGCATCCTTCAT-3′	20	375	60.35
	i23387 lower	5′-CGTCGTCGTTGTACAGTTCC-3′	20		59.21
i11548	i11548 upper	5′-GAGGTGAGCACCATACATGC-3′	20	250	59.12
	i11548 lower	5′-CAAGAAGCCAGAGGATTTCG-3′	20		59.95
i22343	i22343 upper	5′-CATGGCACAGAACAGGCATA-3′	20	297	60.69
	i22343 lower	5′-CTCGGGGACGTGAACAAG-3′	18		60.24
i24572	i24572 upper	5′-ACCATGCGTCACAAAGACAT-3′	20	288	59.01
	i24572 lower	5′-GTCGACCAGTACGGCAACC-3′	19		61.54
QGJ	QGJ upper	5′-GATGAACATGTCCGTGCCTG-3′	20	156	59.27
	QGJ lower	5′-GGTCGCATCGGATAGAGTGT-3′	20		59.33
QGJ (+ intron)	QGJ+Iupper	5′-AAACAGCATGGTGCAGTCAA-3′	20	197	60.31
	QGJ+I lower	5′-TCAGGTGGACAATTGATGAGA-3′	21		59.07
TGS1	TGS1 upper	5′-ACGTTCTTGTGTTGGCATTT-3′	20	180	59.00
	TGS1 lower	5′-TGCTCATCCACAGAAGATGG-3′	20		59.00
ß-tubulin	ß-tubulin upper	5′-GTGGAGTGGATCCCCAACAA-3′	20	158	63.23
	ß-tubulin lower	5′-AAAGCCTTCCTCCTGAACATGG-3′	22		65.85
miR2275a	miR2275a-SLp	5′-GTCGTATCCAGTGCAGGGTCCGAGGTATTCGCACTGGATACGACTGAGAT-3′	50	--	--
	miR2275a-Fp	5′-AACACGCTTTGGTTTCCTCC-3′	20	73*	59.55

miR168	miR168-SLp	5′-GTCGTATCCAGTGCAGGGTCCGAGGTATTCGCACTGGATACGACGGTCCC-3′	50	--	--
	miR168-Fp	5′-AACAAGTCGCTTGGTGCAG-3′	19	72*	59.54
miRNA-Rp universal		5′-GTCGTATCCAGTGCAGGGT-3′	19	--	59.68

*size of the product generated by combination with miRNA-Rp universal primer
